# Biomonitoring of polycyclic aromatic hydrocarbons in firefighters at fire training facilities and in employees at respiratory protection and hose workshops

**DOI:** 10.3389/fpubh.2023.1277812

**Published:** 2023-12-13

**Authors:** Stephan Koslitz, Birgit Heinrich, Heiko U. Käfferlein, Holger M. Koch, Tim Pelzl, Katrin Pitzke, Daniel Köster, Tobias Weiß, Volker Harth, Thomas Brüning, Thomas Behrens, Dirk Taeger

**Affiliations:** ^1^Institute for Prevention and Occupational Medicine of the German Social Accident Insurance, Institute of the Ruhr University Bochum (IPA), Bochum, Germany; ^2^Institute for Occupational Safety and Health of the German Social Accident Insurance (IFA), Sankt Augustin, Germany; ^3^Department of Fire Services, Rescue Services, and Fire Protection of the German Social Accident Insurance, German Social Accident Insurance Institution for the Public Sector in Baden-Württemberg, Stuttgart, Germany; ^4^Institute for Occupational and Maritime Medicine (ZfAM), University Medical Centre Hamburg-Eppendorf (UKE), Hamburg, Germany

**Keywords:** firefighting, workplace, PAH, exposure, occupational hygiene, urine

## Abstract

**Introduction:**

Polycyclic aromatic hydrocarbons (PAHs) are carcinogenic to humans and are formed by incomplete combustion. PAHs are always present during firefighting operations, and fire department members can be exposed to them in the workplace.

**Methods:**

In this study, we analyzed 1-hydroxypyrene (1-OHP) in 36 urine samples from nine firefighters, collected before and after fire training sessions, and 32 urine samples from eight employees at respiratory protection and hose workshops. To assess breakthrough PAH exposure through personal protective equipment and potential dermal uptake, some of the workshop employees wore cotton garments under their regular workwear. Cotton samples were then examined for the presence of 17 semi-volatile and low-volatility PAHs.

**Results:**

After firefighting exercises, we observed approximately a fivefold increase in mean 1-OHP concentrations in samples from firefighters, from 0.24 μg/L to 1.17 μg/L (maximum: 5.31 μg/L). In contrast, 1-OHP levels in workshop employees were found to be low, with the majority of urine samples yielding concentrations below the limit of quantification (LOQ: 0.05 μg/L, maximum: 0.11 μg/L). Similarly, low PAH levels were found on the workshop employees' cotton undergarments, with maximum concentrations of 250 and 205 ng/g for pyrene and benzo[a]pyrene, respectively.

**Discussion:**

In conclusion, significant increases in 1-OHP in urine were observed in firefighters after training sessions, whereas work-related exposure remained low among workshop employees.

## 1 Introduction

Approximately 40,000 full-time and 1.3 million volunteer firefighters in Germany may be exposed to a wide variety of hazardous chemicals during firefighting operations. The compounds formed during combustion depend, among other things, on the burned material, ventilation (oxygen supply), and temperature. Potential hazards include carcinogenic compounds such as polycyclic aromatic hydrocarbons (PAHs), benzene, asbestos, cadmium, or silica (1).

In 2007, firefighting work was classified as potentially carcinogenic to humans by the International Agency for Research on Cancer (IARC) of the World Health Organization (WHO) (Group 2B) (1, 2). A meta-analysis by LeMasters and colleagues (3) provided the basis for this classification. Subsequently, several epidemiological studies on the cancer risk of firefighters were published, including additional meta-analyses (4, 5). These studies reported an increase in overall cancer incidence and in mortality of certain cancers, such as melanoma of the skin, prostate cancer, and mesothelioma. However, the studies showed great heterogeneity in their results. In addition, time- and country-specific effects were also observed (5). Based on the most recent data, IARC re-classified in 2023 occupational exposure as a firefighter as “carcinogenic to humans (Group 1) based on sufficient evidence of cancer in humans” (6, 7). Exposures potentially causal for increased cancer risks, such as PAHs, asbestos, and solar UV radiation, were also mentioned. Robustness of results was observed across sensitivity analyses on mesothelioma and bladder cancer (8).

The majority of human biomonitoring studies to date have dealt with exposures during fire training situations (9–14), although data are still limited. Only one study has been conducted in Germany (14). In addition, exposure of employees who clean contaminated firefighting equipment, in particular, respirators and hoses, has not yet been investigated. Compared to firefighters, employees in workshops are less involved in active firefighting and often do not always wear any personal protective equipment (PPE) that prevents the uptake of hazardous substances. Therefore, we considered workshop employees at fire stations to be an important group of workers who could be exposed to hazardous substances such as PAHs during the cleaning of contaminated firefighting equipment.

## 2 Methods

### 2.1 Study participants and exposure scenarios

Members of the fire brigades of Berlin and Hamburg were invited to participate in the study. In addition to active firefighters, employees of the respiratory protection and hose workshops, and emergency workers at a training facility in Berlin were also included. This cross-sectional study was conducted between 2018 and 2020. In a previous publication, we reported results on firefighters who participated in real-life firefighting scenarios, such as building and car fires (15). In this study, we present data on firefighters who participated in firefighting exercises (*N* = 9) and who were employed in the respiratory protection and hose workshops (*N* = 8); these data were not part of the previous publication (Table 1). Trainee firefighters and workshop employees were informed of the aim and scope of the study on-site and gave written informed consent. The study was approved by the ethics committee of the Ruhr University Bochum, Germany (IRB 17-6071).

**Table 1:** Characteristics of the study population.

**Characteristics**	**Fire training facilities**	**Workshops**
Women	1	1
Men	8	7
Age (mean, min–max)	30.6 (20.4–41.2)	41.4 (26.5–53.0)
Years in fire department (mean, min–max)	6.4 (1.8–15.8)	13.9 (0.8–28.2)
Current non-smokers	4 (44.4 %)	7 (87.5 %)
Current smokers	5 (65.6 %)	1 (12.5 %)

The training scenarios studied consisted of classical flashover training in a container in an enclosed space with high smoke density. The training fire was generated by burning wood. Due to the high smoke scenario, all firefighters wore a self-contained breathing apparatus (SCBA) and standard personal protective equipment that included gloves, fire hoods, and helmets. There were two different roles during these exercise sessions: trainer and trainee. Trainers, typically skilled firefighters, stayed longer in the container than trainees. A training session for the instructors generally lasted 180 min, with ~90 min of direct fire/smoke exposure, whereas the duration of exposure for trainees was much shorter, i.e., 60 and 30 min, respectively. Overall, the training situation of the firefighters, although not completely identical with regard to the burning material and ventilation conditions, can be best compared to that of an attack squad in a fully developed building fire inside a building.

The workshop employees mainly cleaned contaminated SCBAs and dirty hoses that were brought back by the firefighters from training exercises or from fighting real fires such as building and car fires. Usually, the contaminated equipment was first stored outside in a closed container before being brought into the room for cleaning. No protective measures were taken by workshop employees other than the voluntary use of gloves or regular work coats. Frequently, the equipment was inserted directly into the cleaning machine by hand without further pre-cleaning.

### 2.2 Urine collection and analysis

A urine sample was collected from each participant at an initial appointment with the fire station physician (“baseline sample”). The samples were frozen at −20°C and stored until analysis. A self-administered questionnaire was administered consisting of questions on potential co-exposures to PAHs, including, among others, smoking habits and diet. A bag with additional urine containers and an additional questionnaire to store at the workplace was handed out.

The nine firefighters who participated in the training provided three urine samples each: these were provided 2, 6, and ~14 h after training. Together with the baseline samples, a total of 36 samples were collected. The eight workshop employees were also asked to collect three urine samples each after finishing work. Because the workshop employees were assumed to have continuous exposure during their entire work shift, they collected a urine sample 2 h after finishing work and additional urine samples before going to sleep and the following morning. However, the final number of samples was 30 (two samples, each at the third sampling time, were not provided). In general, the recommended time point for biomonitoring of work-associated PAH exposure in terms of 1-OHP is directly after the shift (16). We additionally chose “late sampling time points” (6 and 14 h and pre- and post-sleep, depending on the group) because potential dermal exposure might lead to the delayed uptake of PAHs and excretion of PAH metabolites in urine.

Urine samples were aliquoted and analyzed for 1-OHP as previously described (17). The limit of quantification (LOQ) was 0.05 μg/L of 1-OHP in urine. The coefficient of variation was < 5%. External quality assurance was performed by successful participation in the German External Quality Assessment Scheme for analyses in biological materials (G-EQUAS) (18).

Creatinine was determined based on the Jaffé method (L.u.P. GmbH Labor- und Praxis Service, Bochum, Germany). Creatinine levels between 0.3 and 3.0 g/L are usually considered normal for regularly hydrated persons, whereas urine collection and biomonitoring should be repeated when creatinine levels outside this range are observed (19). However, in the case of the trainee firefighters, we observed creatinine concentrations of up to 4.0 g/L. Because sufficient hydration was difficult to achieve during firefighter training and all firefighters were well-trained individuals with a high muscle-mass-to-body-weight ratio, we chose to include all urine samples to calculate creatine-adjusted 1-OHP levels.

### 2.3 Interpretation of biomonitoring results

For exposure and risk assessment of urinary 1-OHP levels, the Biological Exposure Index (BEI^®^) of the US-American Conference of Governmental Industrial Hygienists (ACGIH) was used. The guidance value of 2.5 μg/L urine does not differentiate between smokers and non-smokers and is a health-based guidance value (20). The BEI^®^ generally indicates a concentration below which nearly all workers should not experience adverse health effects, i.e., in case of PAH exposure and mutagenic (DNA-damaging) effects.

As a second guidance value, the biological reference value (BAR) of the Permanent Senate Commission for the Investigation of Health Hazards of Chemical Compounds in the Work Area (MAK Commission) of the Deutsche Forschungsgemeinschaft (DFG) was used. This guidance level of 0.3 μg/g creatinine is valid for non-smokers only and is not health-based (21). The BAR describes the background level of 1-OHP (in terms of the 95th percentile), which is present in a reference population of people of working age who are not occupationally exposed to PAHs.

Because there is no BAR for smokers, the 95th percentile among smoking individuals from the general population of the 1998 Environmental Survey in Germany (22) was used to interpret the biomonitoring results for smokers (0.7 μg/g creatinine).

### 2.4 Assessing potential skin contamination with PAHs

To assess the potential for PAH contamination of the skin, workshop employees were offered the use of cotton undergarments underneath their regular workwear. For this purpose, cotton gloves (Würth, Künzelsau, Germany) and cotton shirts (HessNatur, Butzbach, Germany) were provided. The gloves and shirts were checked for the absence of PAHs prior to use. The LOQs were between 2.5 ng (for anthracene) and 50 ng (for naphthalene) (for details, see **Table 3**). Four out of the eight study participants wore nitrile gloves, and two of these also wore cotton gloves under their nitrile gloves. One person wore only cotton gloves. The remaining three employees wore no gloves at all. Cotton shirts were worn by three employees.

After use, the cotton shirts and gloves were dried at room temperature, packed carefully to prevent cross-contamination, and stored at −20°C. Under this approach (which was adopted for practical reasons), considerable losses of volatile (i.e., low molecular weight) PAHs could not be avoided. Therefore, only analytical results for benzo[e]pyrene and higher were considered valid.

**Figure 1:**
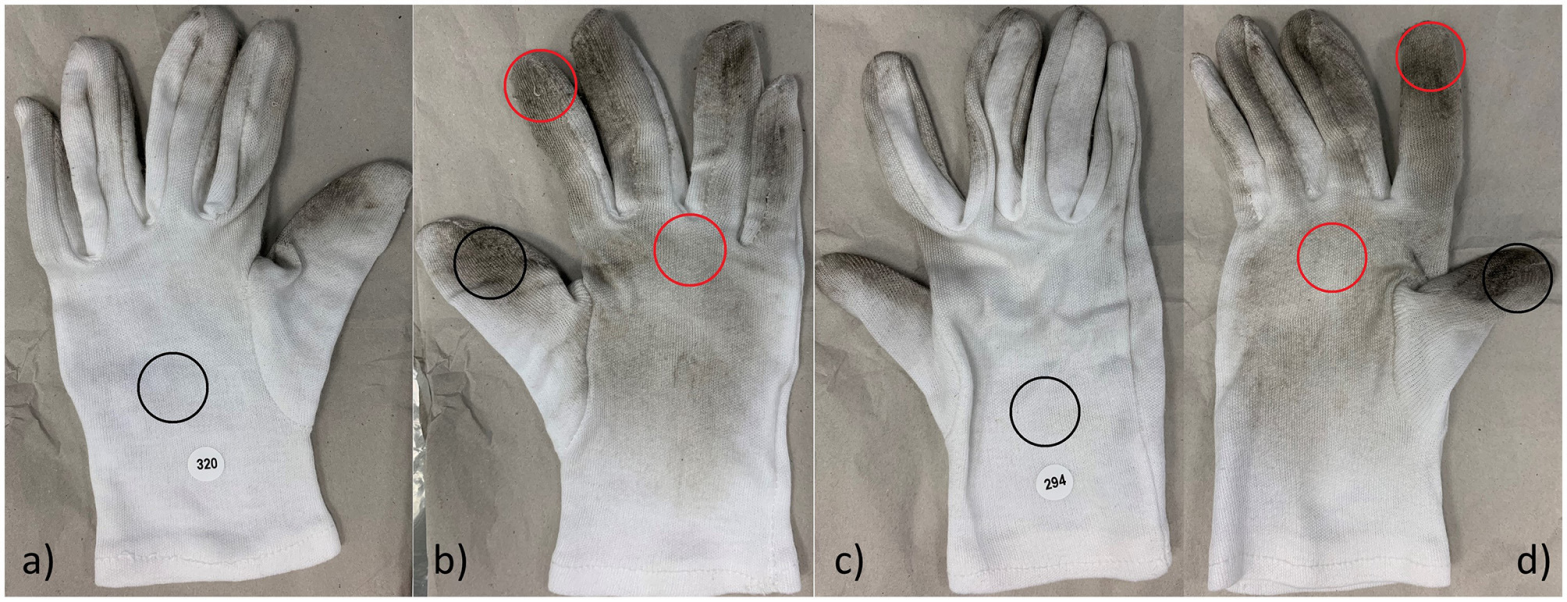
Standardized punch areas on the cotton gloves of the left **(a, b)** and right **(c, d)** hands. The black circles represent pre-selected punch sites defined prior to starting the study; the red circles represent post-selected punch sites with potential additional exposure hotspots, characterized by visible contamination with soot. Please note that the “darkness” of the stain is not a proxy for PAH contamination (see “Results”).

For sample preparation, a standardized punch (diameter 35 mm, Hoffmann SE, Germany) was used. For example, an area of 9.6 cm^2^ was punched out of the gloves both at predefined points and at certain hotspots that were visibly contaminated by soot (Figure 1) and analyzed for PAHs as previously described (23). In brief, the cotton pieces were first weighed to take varying fabric thicknesses and seams into account. Then, the samples were mixed with 2.5 mL of acetonitrile/methanol (60/40 v/v), treated for 60 min in an ultrasonic bath, and shaken on a laboratory shaker. The filtered extracts (PTFE) were finally analyzed using high-performance liquid chromatography coupled with diode array and fluorescence detection (HPLC/DAD-FLD). Concentrations of 16 PAHs from the US Environmental Protection Agency (EPA) list, plus benzo[e]pyrene, were determined (24). Naphthalene, acenaphthylene, and acenaphthene were detected by DAD; the other compounds were detected by FLD. The coefficient of variation was < 5%. Analytical results are presented in ng/g fabric.

### 2.5 Interpretation of cotton results

To interpret the PAH concentrations in the punched cotton pieces (EU), Regulation 2018/1513 was used (25). This regulation describes the current EU restrictions on the manufacture, sale, and use of selected carcinogens, mutagens, and reproductive toxicants (category 1A, 1B) in clothing and related accessories, including textiles and footwear. Currently, maximum values of 1 ppm (= 1 mg/kg = 1.000 ng/g) in new clothing materials are enforced for benz[a]anthracene, chrysene, benzo[e]pyrene, benzo[b]fluoranthene, benzo[j]fluoranthene, benzo[k]fluoranthene, benzo[a]pyrene (B[a]P), and dibenz[ah]anthracene.

### 2.6 Statistical analysis

Descriptive statistics were used to characterize 1-OHP concentrations at the four sample time points (baseline plus three post-event time points) and PAH measurements in the cotton samples. Because of the lack of normal distribution of the measurements, the median and the arithmetic mean, the minimum, and the maximum were calculated. Concentrations were plotted against time points for each participant. The non-parametric Wilcoxon matched-pairs signed-rank test was used to compare the median levels of 1-OHP occurring after the shift to those measured at baseline. The software package SAS version 9.4 (SAS Institute Inc, Cary, NC, USA), was used for analyses. For graphs, GraphPad Prism Version 9.5.0 (GraphPad Software, Boston, USA) was employed.

## 3 Results

### 3.1 Firefighters at training facilities

**Figure 2:**
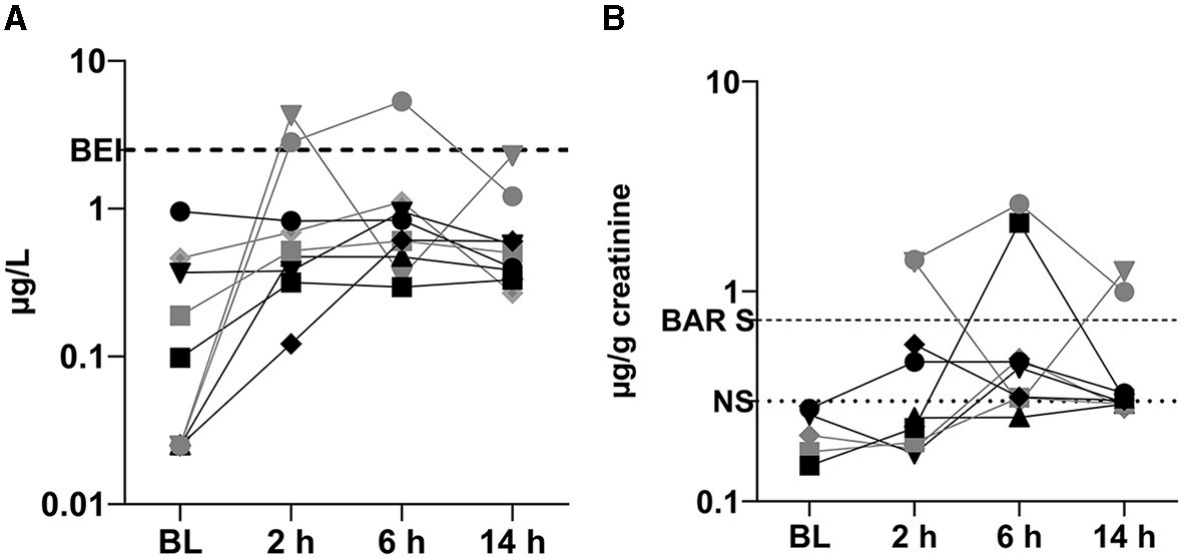
Volume-related **(A)** and creatinine-related **(B)** 1-OHP concentrations before and after firefighting training sessions, classified by smoking status (black symbols: smokers; gray symbols: non-smokers). BL, baseline measurement.

1-OHP concentrations in the baseline urine samples were generally low (maximum: 0.96 μg/L or 0.28 μg/g creatinine) and within the range of the BAR levels of non-smokers (Table 2). After training, 1-OHP levels were above the LOQ in the majority of cases (25 out of 27 cases). Two urine samples from two different firefighters (4.30 μg/L and 5.31 μg/L) exceeded the BEI^®^ level for 1-OHP of 2.50 μg/L. Of note, both firefighters were non-smokers, and their respective baseline samples were below the LOQ. One of the firefighters, an instructor who used an SCBA for 120 min during the training session, showed an unusual pattern of 1-OHP excretion: 1-OHP concentration exceeded the BEI^®^ at sampling point 1 (4.30 μg/L), then dropped considerably (0.35 μg/L), and almost reached the BEI^®^ level again at the third sampling point (2.30 μg/L) (Figure 2A). This pattern remained after creatinine correction (1.38, 0.29 and 1.25 μg/g creatinine). Interestingly, the remaining firefighters, some of whom worked up to 180 min using an SCBA, showed no increase beyond the BEI^®^.

**Table 2:** Summary of urinary 1-OHP measurements (*N* = 17).

**Parameter**	**1-OHP (**μ**g/L)**						**1-OHP (**μ**g/g creatinine)**		
	***N***	**N (>LOQ)**	**Mean**	**Median**	**Range**	***P*-value^‡^**	**Mean^†^**	**Median^†^**	**Range^†^**
**Fire training facilities**									
Baseline	9	5	0.24	0.10	< LOQ^*^–0.96		0.21	0.21	0.15–0.28
1st sampling	9	8	1.16	0.52	0.12–4.30	0.0195	0.53	0.24	0.17–1.42
2nd sampling	9	8	1.17	0.61	0.29–5.31	0.0078	0.64	0.37	0.25–2.61
3rd sampling	9	9	0.73	0.50	0.27–2.30	0.0742	0.48	0.30	0.28–1.25
**Workshops**									
Baseline	8	2	< LOQ	< LOQ	< LOQ−0.12		-	-	-
1st sampling	8	3	< LOQ	< LOQ	< LOQ−0.11		-	-	-
2nd sampling	8	4	0.05	< LOQ	< LOQ−0.10		0.06	0.06	0.03–0.08
3rd sampling	6	2	0.05	< LOQ	< LOQ−0.10		-	-	-

^*^ If values for volume-related levels were < LOQ (0.05 μg/L), $\frac{1}{2}$ LOQ was used for statistical analysis; only volume-related levels >LOQ were corrected for creatinine.^‡^p-values for the Wilcoxon matched-pairs signed-rank test compared to baseline results.^†^If no values are reported, ≤ 3 volume-related 1-OHP values were >LOQ.

When evaluating the creatinine-corrected concentrations, in 55.6% of the firefighters (five out of nine), the respective reference level for the firefighter (smoker or non-smoker) was exceeded by up to eight-fold, thus suggesting firefighting-associated exposure to PAHs (Figure 2B), whereas the remaining four firefighters remained within the respective reference level for smokers or non-smokers.

### 3.2 Employees at workshops

Compared to those of the firefighters after training sessions, 1-OHP concentrations measured in the workshop employees were much lower. All 1-OHP measurements were below the BEI^®^ and below the respective BAR for smokers or non-smokers, depending on the participant's smoking status.

Almost all samples (six out of eight) were below the LOQ at the baseline time point. Even after the employees had completed their cleaning tasks, 59% (13 out of the 22 post-shift samples) remained below the LOQ, thus leading to mean and median concentrations at or below the LOQ. The maximum observed concentration was 0.12 μg/L, which was approximately twice the LOQ. This value was observed in a baseline urine sample. Of the two workshop employees with 1-OHP values above the LOQ at baseline, one was a smoker (0.12 μg/L) and the other was a non-smoker (0.06 μg/L) who reported having eaten smoked and grilled products in the 24 h before urine sampling.

Three of the seven workshop employees provided gloves that they had worn, and in total, 24 cotton pieces were analyzed. For two of the three workshop employees, all PAH levels provided in the samples were below the respective LOQs. However, a wide range of PAH levels was quantified in the eight punch samples from the pair of cotton gloves provided by the third workshop employee (Table 3), who was wearing only cotton gloves during work (with no additional nitrile gloves over them). Therefore, the PAHs found in the cotton material would have been on the employee's hands if he had worked without gloves.

**Table 3:** Summary of PAH concentration for one pair of cotton gloves (see also Figure 1) from the respiratory protection and hose workshops (*N* = 8).

**PAHs**	**LOQ [ng/punch sample]**	**N > LOQ**	**Mean [ng/g]**	**Range (min-max) [ng/g]**
Naphthalene	50	0	-	-
Acenaphthylene	50	0	-	-
Acenaphthene	25	0	-	-
Fluorene	25	0	-	-
Phenanthrene	13	7	184	76–398
Anthracene	2.5	5	22	11–44
Fluoranthene	11	6	136	85–261
Pyrene	5.0	7	131	34–250
Benz[a]anthracene	3.8	6	101	52–170
Chrysene	3.8	6	95	42–170
Benzo[e]pyrene	25	4	153	131–182
Benzo[b]fluoranthene	5.0	6	107	34–172
Benzo[k]fluoranthene	4.5	5	71	34–100
Benzo[a]pyrene	4.3	6	133	37–205
Dibenz[ah]anthracene	7.3	0	-	-
Benzo[ghi]perylene	4.3	5	112	53–159
Indeno[1,2,3-cd]pyrene	7.5	5	119	57–159

Sixteen EPA-PAH compounds were measured; gray shading indicates selected PAH compounds that are also regulated by EU regulation 2018/1513 (maximum levels allowed in new textiles marketed in the EU: 1 ppm = 1 mg/kg = 1.000 ng/g; benzo[j]fluoranthene was not measured).

The samples included the well-accepted carcinogen benzo[a]pyrene (37–205 ng/g) and pyrene, which is not known to be carcinogenic (34–250 ng/g). The latter is the parent compound of 1-OHP in urine, which was detected in the employee at regular background levels. Generally, the punch pieces taken from the back of the hands had lower contamination levels than the samples from the thumbs, the hotspots on the palms, and especially the index fingers. The highest concentration levels were observed in the left index finger (Figure 1b). Of note, all concentrations were a factor of 4–5 below the level allowed by (EU) Regulation 2018/1513 for new textile products (25).

All three subjects who wore cotton gloves, also provided cotton shirts. The PAH levels in all 18 punch samples that were taken from the shirts were below the respective LOQ.

## 4 Discussion

Trainee firefighters and workshop employees showed significant differences in their exposure to PAHs. Whereas, firefighters exhibited an almost five- to sixfold increase in mean urinary 1-OHP concentration (in μg/L) after a shift compared to the baseline measurements, employees in the workshop were not occupationally exposed to PAHs. The latter exhibited baseline as well as post-work 1-OHP levels that were clearly within the respective reference values for smokers and non-smokers in the general German population.

Despite similar exposure settings due to the use of standardized training procedures, we observed a wide range of 1-OHP levels in urine after the training sessions, although the variability was less pronounced for creatinine-normalized levels (0.17–2.61 μg/g creatinine) compared to volume-related levels (0.12–5.31 μg/L). Furthermore, all firefighters wore similar personal protective equipment as they were all part of the same fire brigade and were equipped with the same PPE. There were some differences between firefighters in terms of the amount of time for which the SCBA was worn, although this did not affect internal exposure levels (data not shown).

Generally, our results are in line with those of previous studies after firefighting training in various countries (9–14). In these studies, a wide range of exposure levels (mostly presented in volume-related levels, μg/L) was observed. The majority of studies observed approximately a two- to sevenfold increase in 1-OHP levels after training sessions (9, 11–14) and, in particular, after burning of chipboard in containers, which is in line with our findings. In addition, a two- to threefold increase in the levels of other hydroxylated PAH in urine samples, such as OH-naphthalenes, OH-fluoranthene, and OH-phenanthrenes, was also observed (14). Interestingly, when diesel was used to burn fires in containers as well as in barrels, no significant increase in 1-OHP could be observed (9, 13). In contrast, increases in 1-OHP levels by up to 30-fold were observed in firefighting trainers when conducting several fire training exercises in a row (i.e., three fire training exercises per day) (10), thus indicating that increased numbers of fire training exercises in a short period of time may result in increased PAH exposure levels.

Although the differences are most likely negligible, the increases in the 1-OHP levels of firefighters conducting training exercises, as reported here, and those in previous studies appear slightly higher compared to those that have been reported in firefighters after real fire missions (15, 26–28). There, only a two- to threefold increase in 1-OHP levels has been observed. These slight differences also became apparent when evaluating the frequency of BEI^®^ exceedance. Of the nine firefighters in our study, two exceeded the BEI^®^ (one of them at two sampling points). Comparing this to our previously published study on firefighters in real firefighting missions (15), we also observed two instances of BEI^®^ exceedance, but this was among a total of 77 firefighters. Reasons may include slightly varying exposure circumstances, such as greater distances when extinguishing real fires or in the presence of fully deployed fires. Therefore, 1-OHP levels in firefighters conducting training sessions are more similar to those of attack teams in the field, i.e., firefighters getting close to flames and smoke in fighting fires where respiratory protection is needed.

The observed increase in 1-OHP in our study was less pronounced (about twofold) after adjustment of the levels by creatinine. However, because the majority of previous studies reported volume-related concentrations, no direct comparison was possible. We recommend that results should be presented as both volume- and creatinine-related levels to better compare results between studies. In addition, in presenting biomonitoring results for subjects with a high muscle-to-body-mass ratio (such as firefighters) creatinine correction seems reasonable.

The 1-OHP levels in the urine of firefighters (either after training or after fighting real fires) appear low relative to those of industrial workers (29). These lower exposure levels became particularly evident when comparing creatinine-normalized values. Median 1-OHP levels in our study after training exercises (0.37 μg/g creatinine) and in our previous study (15) investigating firefighters after real firefighting missions (0.12 μg/g creatinine) were ~10- to 100-fold lower compared to those in industrial workers, i.e., workers employed in the production of coke (3.8 μg/g creatinine), refractory materials (8.4 μg/g creatinine), carbon electrodes (9.7 μg/g creatinine), and steel (13.5 μg/g creatinine). Even the maximum 1-OHP concentration observed in our study (2.61 μg/g creatinine) was lower than the median concentrations in workers at the aforementioned industrial workplaces (29). Nonetheless, despite the short time of exposure and the use of protective equipment, the slightly increased levels of 1-OHP in firefighters above normal background levels are evidence of firefighting-associated exposure to PAHs. The differences in internal exposure between firefighters and industrial workers, next to differences in external exposure levels, are most likely caused by the use of special protective equipment (including SCBA). Compared to firefighters, industrial workers usually wear, if they use any PPE at all, dust masks (FFP3), overalls, and leather gloves.

Although this interpretation is speculative because direct evidence is missing, PAH exposure in firefighters occurs most likely via dermal uptake. First, personal protective equipment, including SCBA, was frequently used; thus, inhalation exposure during firefighting can be excluded almost with certainty. Second, and in line with other studies (10, 12–14), the peaks of 1-OHP excretion in the urine of firefighters were always slightly delayed, i.e., they occurred 4–6 h after finishing the training or after real fire missions (i.e., the at second sampling point) (15). These findings are in line with delayed absorption, metabolism, and excretion of PAH after dermal uptake. Interestingly, current regulatory guidelines for assessing PAH exposure in terms of 1-OHP in urine recommend urine collection directly after the end of the work shift. However, these guidelines pertain specifically to respiratory exposure routes. In our investigation, where dermal absorption is the major route of exposure, biomonitoring directly after a shift may underestimate exposure levels.

In contrast to firefighters, PAH exposure of employees who clean contaminated firefighting equipment, in particular respirators and hoses, has not previously been investigated. We were able to demonstrate that PAHs were clearly present in the work environment in terms of contaminated equipment. For example, we detected a wide range of semi-volatile and low-volatility PAHs in the punched cotton glove samples of a worker who had worked with contaminated firefighting equipment. Interestingly, based on the REACH regulation for marketing new clothes on the European market, the gloves still could have been sold on the market (25). The observed amounts of seven selected PAH compounds that are regulated by the guidelines were, in each case, below the current EU threshold value of 1 mg/kg (=1,000 ng/g) (Table 3). Nonetheless, the general validity of this finding is certainly limited due to our measurements having been obtained in only a single pair of gloves. The extent of contamination is most likely different each day and might strongly depend on where the equipment was used during the previous firefighting operation. However, because respiratory protection and hose workshops are operated centrally for fire stations, the materials of several firefighting operations are usually cleaned in a single day. Therefore, the PAH residues found on the gloves in our study may also have been derived from contaminated equipment that has been previously used by firefighting attack teams.

Of utmost importance, four of the eight workers in the workshop wore gloves (cotton and/or nitrile gloves). Therefore, it is not surprising that no work-related internal exposure to PAHs, in the form of increased urinary 1-OHP, was observed in the workshop employees. In the majority of cases, 1-OHP levels were below the LOQ, and the maximum observed concentration (0.12 μg/L) was more than 20 times lower than the BEI^®^. Moreover, depending on the smoking status of the employees, no exceedance of the respective reference values for smokers or non-smokers was recorded. The results suggest that work-associated dermal uptake of PAHs present in the work environment could be almost completely avoided. Therefore, reducing internal work-related exposure can be successfully achieved by wearing gloves.

A major strength of our study is that the internal exposure to PAHs was measured in terms of 1-OHP, i.e., the amount of PAH that was actually taken up by firefighters and workshop employees was examined. Our results show that, irrespective of the presence of PAHs during fires or on contaminated firefighting equipment, protective clothing is highly efficient in minimizing the uptake of PAH. By using simple cotton gloves, we were also able to show that significant contact with PAHs can occur in employees of respiratory protection and hose workshops. Therefore, the use of such gloves is clearly recommended.

The limitations of the study include the fact that the study population was rather small and was not a random sample of firefighters at fire training facilities and workshop employees. Another limitation is that the specific working tasks of workshop employees and the actual contamination of the firefighting equipment remain unknown. There also might have been exposure by inhalation to volatile PAHs that might have been missed by measuring 1-OHP in urine.

## 5 Conclusions

By using a biomonitoring approach, we showed that using personal protective equipment during training sessions (such as SCBA and firefighter clothing) is highly effective in minimizing PAH exposure. The same applies to the wearing of gloves among workshop employees who are responsible for cleaning firefighters' PAH-contaminated protective gear. Overall, compared to industrial workers, exposure to PAHs in firefighters and employees in firefighting-associated jobs such as cleaning protective gear is low. However, due to the limited number of participants involved in our study and the lack of previous studies on workshop employees, the results should be confirmed in a larger study.

## Data availability statement

The raw data supporting the conclusions of this article will be made available by the authors, without undue reservation.

## Ethics statement

The studies involving humans were approved by the Ruhr University Bochum, Germany. The studies were conducted in accordance with the local legislation and institutional requirements. The participants provided their written informed consent to participate in this study.

## Author contributions

SK: Conceptualization, Data curation, Formal analysis, Investigation, Methodology, Project administration, Validation, Visualization, Writing—original draft. BH: Investigation, Methodology, Writing—review & editing. HUK: Project administration, Supervision, Writing—review & editing. HMK: Methodology, Supervision, Writing—review & editing. TP: Conceptualization, Funding acquisition, Project administration, Writing—review & editing. KP: Investigation, Resources, Writing—review & editing. DK: Investigation, Methodology, Writing—review & editing. TW: Resources, Supervision, Writing—review & editing. VH: Investigation, Resources, Supervision, Writing—review & editing. TBr: Project administration, Resources, Supervision, Writing—review & editing. TBe: Funding acquisition, Resources, Supervision, Writing—review & editing. DT: Conceptualization, Funding acquisition, Investigation, Methodology, Project administration, Supervision, Visualization, Writing—original draft.
